# Supplementary material for: Differentiable molecular simulation can learn all the parameters in a coarse-grained force field for proteins
Source: PLoS One. 2021 Sep 2;16(9):e0256990. doi: 10.1371/journal.pone.0256990 (PMC8412298; doi:10.1371/journal.pone.0256990)
Supplement: S1 File — (PDF) [file pone.0256990.s001.pdf]

Differentiable molecular simulation can learn all the parameters in a coarse-grained force field for proteins

Joe G Greener, David T Jones

S1 File

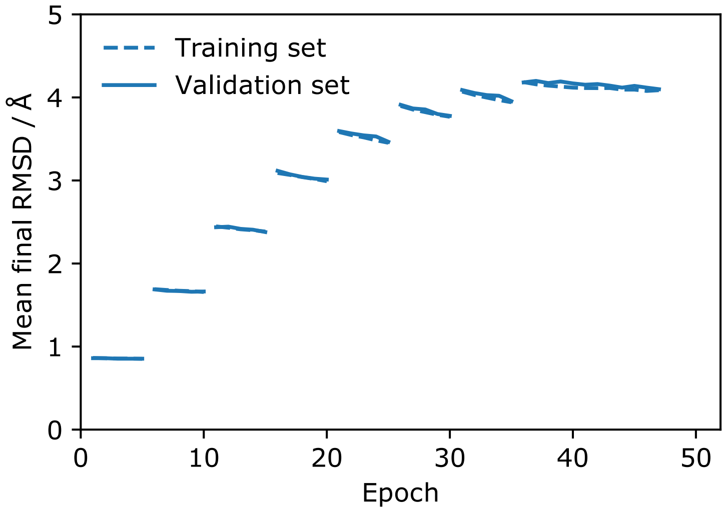

S1 Fig Training progress. The mean final RMSD across all atoms in the coarse-grained model for each epoch is shown for the training set and the validation set. As outlined in the methods the number of steps simulated starts at 250 and increases by 250 every 5 epochs, reaching a maximum of 2,000 steps at epoch 36. At epoch 38 the Adam optimiser was reset with a lower learning rate. The parameters after epoch 45 are used in the results.

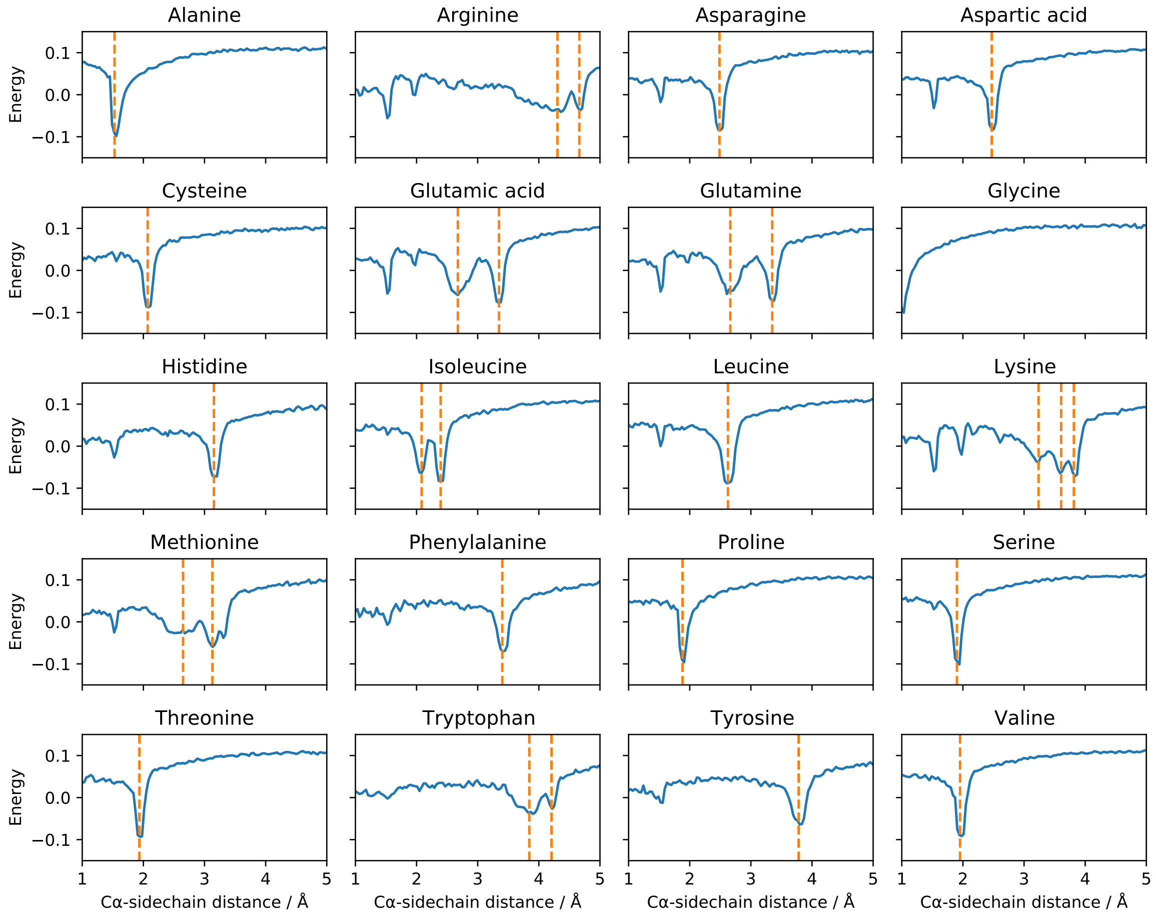

S2 Fig Learned distance potentials between the Cα atom and the sidechain centroid for each amino acid. The orange lines indicate minima in the PDB distributions of these distances, corresponding to different rotamers.

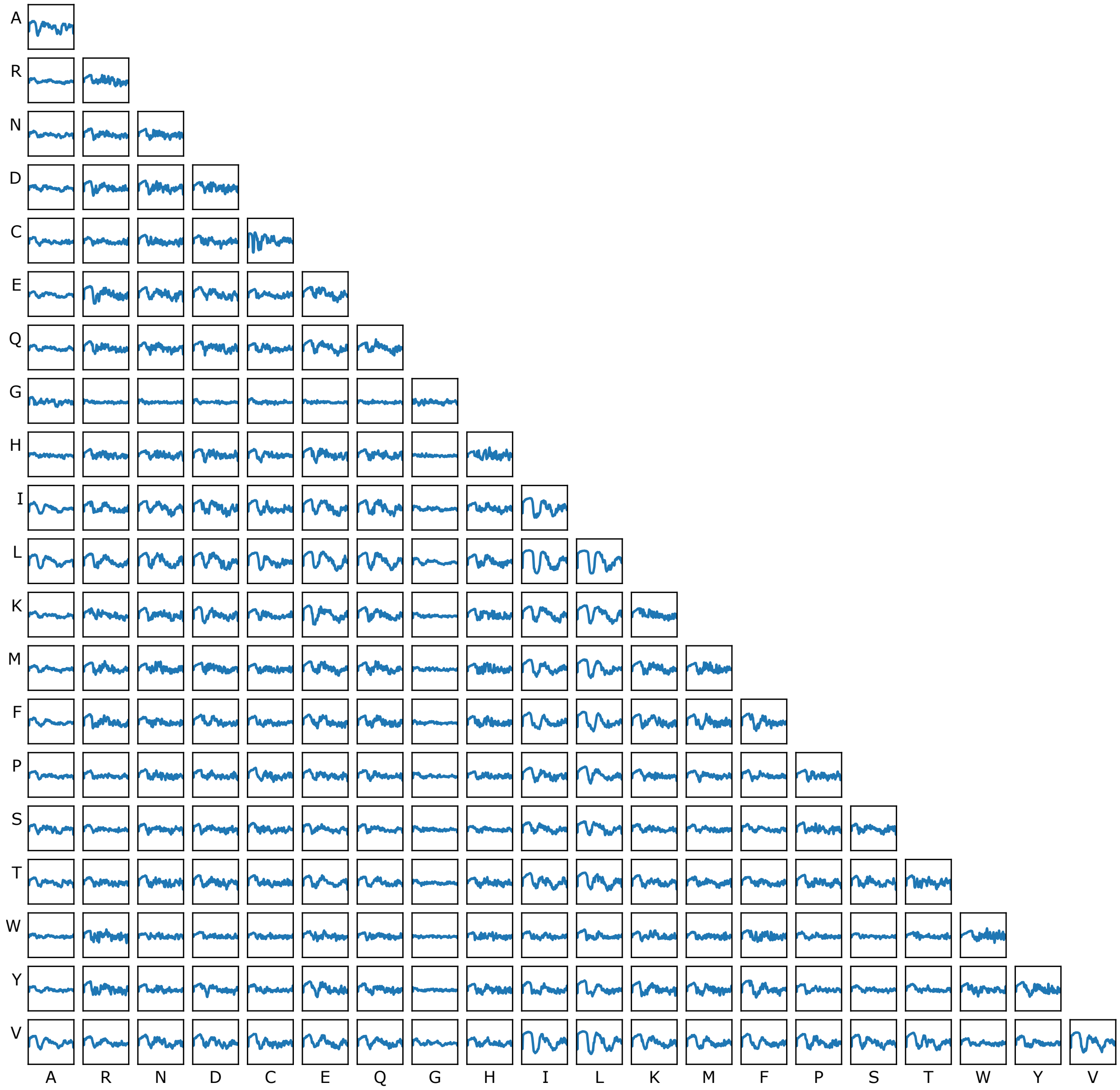

S3 Fig Learned distance potentials between the sidechain centroids for each amino acid pair. On each plot the *x*-axis is the distance between the sidechain centroids and runs from 1 Å to 15 Å. The *y*-axis is the energy in the learned potential and runs from -0.2 to 0.23. There are separate potentials for residues close in sequence, not shown here.

Protein or peptide	PDB ID	Sequence length	Sequence	Predicted secondary structure
Alanine dipeptide	-	2	AA	CC
$(AAQAA)_3$ repeat peptide	-	15	AAQAAAAQAAAAQAA	CHHHHHHHHHHHHHC
Chignolin	-	10	YYDPETGTWY	CCCCCCCCCC
Trp-cage	2JOF_A	20	DAYAQWLADGGPSSGRPPPS	CHHHHHHHCCCCCCCCCCCC
BBA	1FME_A	28	EQYTAKYKGRTFRNEKELRD<br>FIEKFKGR	CCCCCCCCCCCCCCHHHHHH<br>HHHHHCCC
Villin HP36	2F4K_A	35	LSDEDFKAVFGMTRSAFANL<br>PLWLQQHLLKEKGLF	CCHHHHHHHHHCCHHHHHCH<br>HHHHHHHHHHHCCCC
WW domain	2F21_A	33	KLPPGWEKRMSRDGRVYYFN<br>HITGTTQFERPSG	CCCCCHHEEECCCCCEEEEE<br>CCCCCCCCCCCCC
NTL9	2HBA_A	39	MKVIFLKDVKGMGKKGEIKN<br>VADGYANNFLFKQGLAIEA	CEEEEEECCCCCCCCCCEEE<br>CCCCCCCCCHHHCCCEEEC
BBL	2WXC_A	47	GSQNNDALSPAIRRLLAEWN<br>LDASAIKGTGVGGRLTREDV<br>EKHLAKA	CCCCHHHHHHHHHHHHHHCC<br>CCHHHCCCCCCCCCCHHHHH<br>HHHHHHC
Protein B	1PRB_A	47	LKNAIEDAIAELKKAGITSD<br>FYFNAINKAKTVEEVNALVN<br>EILKAHA	CCHHHHHHHHHHHHCCCCCH<br>HHHHHHHHCCCHHHHHHHHH<br>HHHHHCC

S1 Table Details of the proteins and peptides used. The PDB ID and chain ID are given along with the amino acid sequence used for modelling and the PSIPRED single sequence secondary structure prediction [82]. The sequences used for Trp-cage, villin HP36, WW domain, NTL9 and protein B contain mutations and are the same as those used in Lindorff-Larsen et al. 2011 [59]. The chignolin structure was taken from the supplementary data of Honda et al. 2008 [62]. In the cases where the structure is an NMR ensemble (chignolin, Trp-cage, BBA and BBL), the first model in the ensemble was used when calculating RMSD values.
