# Supplementary material for: Differentiable molecular simulation can learn all the parameters in a coarse-grained force field for proteins
Source: PLoS One. 2021 Sep 2;16(9):e0256990. doi: 10.1371/journal.pone.0256990 (PMC8412298; doi:10.1371/journal.pone.0256990)
Supplement: Attachment — Submitted filename: DMS paper responses to reviewers.pdf [file pone.0256990.s002.pdf]

Responses to reviewers

We thank the reviewers for their valuable comments. We have made changes to address the issues and believe that with these changes the paper is ready for publication. In particular we have expanded the fraction of native sequence results to an additional 4 larger proteins, demonstrated that the learned potential is able to achieve native-like sequences in some cases with fixed backbone design, clarified aspects of the method and added additional references.

Reviewer 1

Greener and Jones proposed to use automatic differentiation adapted from deep learning for learning all the parameters for molecular dynamics force field. They examined this method in a simple coarse-grained force field and used the learned force potential to study protein folding, scoring, and design on a set of small proteins. The method is useful and interesting, and the results seem promising. I have the following comments.

We thank the reviewer for their comments.

1. They mentioned that the modeling is trained on a set of 2004 diverse proteins up to 100 residues, but in the case studies (Table S1), the largest protein is only containing 35 amino acids. I wonder how their learned potential performs on larger proteins, e.g., ~100 amino acids, for folding, scoring, and design.

Please see below.

2. The test proteins are too few and small. They should include more and larger proteins for testing.

We have added 4 more proteins used to study similar methods - WW domain, NTL9, BBL and protein B - to the protein design results. See Figure 5 and details of the proteins in Table S1. The results on the new proteins are similar to those on the original set, with a larger native sequence fraction giving a lower energy in the learned potential. We believe that this is sufficient for a study validating a new force field parameterisation approach such as the one presented here. Applying the folding simulations to larger proteins is a topic of future research and may involve the use of enhanced simulation, as is the case in UNRES and CABS-fold. This is mentioned in the manuscript.

3. In the so-called protein design in this work, the authors did not systematically perform de novo sequence designs on fixed backbones. Instead, they evaluated the energy distributions of a set of sequence decoys with varying fraction of native sequence. Besides, they mutated residues according to PDB distributions. I wonder what is the performance of their learned force field for the computational de novo sequence design benchmark, i.e., “native sequence recapitulation rate”? Again, the authors just compared the native sequence and a set of

sequence decoys on a set of mini proteins. This cannot represent a general performance of their useful learned potentials. I want to know 1) the performance of their potential on a larger set of larger proteins and 2) the comparison of their potential on native sequence recapitulation with other protein design approaches such as Rosetta and EvoEF2, just as they did for protein folding to compare with UNRES and CABS-fold.

The original manuscript had fixed backbone design results for chignolin, using a simple procedure of mutating residues and accepting changes based on the change in energy. We have now expanded these results to the set of 8 proteins and compared the results to EvoEF2, which uses an energy function consisting of nine terms and is developed specifically for protein design applications. The results are shown in Table 1. The results from the learned potential are comparable to those from EvoEF2, with our potential performing better on the smaller proteins and worse on the larger proteins. Training the potential specifically for protein design and applying it to larger proteins is a topic of future work.

4. Why NVE for training while NVT for testing? To my knowledge, NPT ensemble is often used for MD.

We used the NVE ensemble for training as there is no thermostat involving random numbers, which could have affected the accuracy of the gradients during automatic differentiation. We did briefly try using the NVT ensemble for training and there were no obvious problems but we proceeded with the programmatically simpler NVE ensemble to better reason about the gradients in the system. In practice the training simulations were up to 2,000 steps, only a fraction of the testing simulations. NVT and NPT ensembles are used for MD but we did not use a conventional periodic bounding box, meaning the coarse-grained system did not have a natural measure of pressure or volume and effectively these ensembles are the same.

5. Some Figures are distractedly discussed in the manuscript, e.g., Figure 3B should be Figure 3C, and some figures/subfigures have never been mentioned at all.

The reference to Figure 3C has been corrected. References to each subfigure have been added to the manuscript.

Reviewer 2

The paper describes an interesting method for force-field optimization, which is based on machine learning. The Authors developed a new coarse-grained model of proteins, in which each residue is represented by 4 interaction sites (N, carbonyl-C, Calpha, and sidechain center). In optimization steps, whole microcanonical MD simulations are performed on the training proteins, starting from their experimental structures, and the potentials are optimized by using the Adam algoritm with automatic derivative calculations, the target function being log(1+rmsd), where rmsd is the root mean standard deviation from the experimental structure. Whole potential curves are determined; hence the potentials depend only on a single distance or angle/torsional angle, no dependence on orientation included. The optimized potentials were

tested only against mini-proteins in de novo folding simulations but this does not seem to be a problem, because the Authors' objective was to demonstrate the principle rather than to produce a force field of practical application at this point. Moreover, the Authors have demonstrated that the potentials perform well in threading with minimization and in inverse folding.

The paper is very interesting and well written. I enjoyed readinig it. However, the following minor points should be addressed before it is accepted for publication:

We thank the reviewer for their comments.

1. Page 15, the "Training" section. Some more details should be given about the optimization procedure, in particular how te Adam optimizer with automatic derivatives works. Only the description of the calculation of the loss (target) function is provided but how are the gradients of the target function calculated? Referring to PyTorch is not a sufficient description.

We have added a description of how PyTorch calculates gradients from a loss function using reverse mode automatic differentiation through a computation graph. We have outlined how Adam uses these gradients to improve the parameters and how Adam differs from gradient descent.

2. How were the simulations with CABS-fold and UNRES-server carried out? The Authors state that both servers use secondary-structure prediction but, by default, both run in the ab initio mode. Therefore, the Authors should state that they input the secondary-structure information. Also, the UNRES server supports three force-field variants: the old FF2 (which is the default), OPT-WTFSA-2 [JCIM, 57, 2364-2377 (2017)] and the latest (and most advanced scale-consistent variant [NEWCT-9P (JCP, 150, 155104 (2019)]. I guess that the Authors used the FF2 variant, but this should be stated. Also, UNRES when run in MREMD mode produces 5 clusters of conformations. Did the Authors include the rank#1 structure or the lowest-RMSD structure in the analysis? Besides, UNRES server can also be run in canonical mode and it provides RMSD along the trajectory, from which the distribution can be extracted, which could be compared with those from the learned potential.

UNRES server models were generated with the FF2 force field, extended chain start, secondary structure restraints, Berendsen thermostat with 1.0 coupling to the thermal bath, 0.02 Langevin scaling of the friction coefficient, and 8 replicas exchanging every 1,000 steps with temperatures ranging from 270 K to 340 K in steps of 10 K. The number of steps was increased to the maximum of 10^7. The top ranked model was used. CABS-fold models were generated with default de novo parameters including CABS temperature 3.5-1.0. The top ranked model was used. The above information has been added to the methods.

3. I am somehow puzzled that so long wall-clock time /10,000,000 steps is required (36 hrs on GPU; page 7, line 14 from the bottom). For 1,000,000 steps with the BBA mini-protein, UNRES server required 900 secs. wall-clock time, which translates to 2,5 wall-clock hrs per 10,000,000 steps on a single INTEL core (no GPU use). From my experience, CABS is comparable in timing or even faster (unfortunately, the CABS server was not functioning properly at the time I

was writing this review). Model complicacy seems to comparable; CABS has 3 interaction sites (Calpha, Cbeta SC) and UNRES 2 (peptide groups and SC, but more complicated potentials) and, therefore, some optimization might be missing in force calculation. One thing that could be improved would be to store the numerical derivatives of the potentials in distance in addition to the potentials; this could save one subtraction and one division (point 3 in page 14). Also, symmetric divided differences could be used to improve the accuracy of the forces.

The code has to be implemented in such a way that PyTorch can calculate gradients, i.e. with array operations and no element-wise mutations. There are certainly optimisations that could be made to speed up the code, however that was not a primary focus of the study. In particular, it would be possible to write a different implementation for inference that is not constrained in the above way and hence is faster, or to load the parameters into an existing simulation framework. However, for the study in question we find that the < 15 ms taken per step is acceptable. As stated in the manuscript it is ~3x slower on CPU, indicating non-optimal GPU usage but also showing that running on CPU is a viable option.

4. The description of the optimization procedure suggests that the experimental structures of the training proteins are only perturbed by running MD in the NVE mode. An immediate concern is that the potentials obtained that way will be biased towards the experimental structures. The fact that there are many training proteins probably makes this concern less serious but the Authors should provide more discussion about the transferability problem (both to non-native states and to other proteins). A maximum-likelihood approach, in which non-native structures are taken into account is described in refs. 37 and 38; also, there are recent papers by the D.E. Shaw group, in which they parameterize the all-atom force field to handle intrinsically-disordered proteins.

It is correct that we only train the model by perturbing the native structure with MD. It is possible that the learned potential would favour folded structures, which is a general problem faced by most force fields which have been parameterised over many years to fold proteins and keep them folded. We do notice that increasing the temperature of the thermostat when collecting results leads to unfolded and variable structures, providing some evidence that there is not a heavy bias towards globular structures. We have mentioned this in the manuscript. For this study we hope that the large training set of 2,004 diverse proteins and the lack of homology between the training set and test set mean that there is no bias towards particular structures, which we have tried hard to avoid and which affects related machine learning studies. We intend to use this work as the basis to explore further differentiable simulations targeting loss functions appropriate to intrinsically disordered structures such as radius of gyration and high flexibility. We have mentioned these possibilities in the discussion.

5. Figure 4. The superposition of the structure of villing headpiece obtained with the learned potentials does not seem to have the RMSD of 7.38 A. It rather looks like the perturbed native structure with about 2 A RMSD (see the left part of the panel that shows the RMSD distributions). Also, the fact that no conformation obtained in the simulation of villin started from the structure generated with secondary-structure prediction reached 12 low RMSD in 12 million steps raises concern. On the other hand, the simulation started from the experimental structure

did not leave the native basin (the lef-bottom panel of Figure 4). If this simulation also lasted 12 million steps, the ergodicity of the simulations is of concern. Perhaps more shorter trajectories should be run.

The villin superposition was not displayed clearly in Figure 4; it is a 7.38 Å model but the N-terminal helix faces the wrong way. The image has been rotated to make this clearer. It is true that the simulation parameters we use do not explore the complete conformational landscape, in this case the villin reaching and staying at a low energy state which is topologically similar to the native structure but with one helix pointing the wrong way. By comparison, villin remains in a native basin when the simulation starts from the native structure. As we mention later in the results, simulating at a higher temperature is able to reach a 4.19 Å model but we wanted all the simulations in Figure 4 to share the same parameters for consistency and transparency. A mention of the above has been added to the results. UNRES, CABS-fold and similar methods often use enhanced sampling approaches to better explore the conformational landscape and we intend to look at this in future work.

6. For the reader's benefit, the Authors could mention other approaches at force-field optimization, including those of Crippenn and colleagues and Wolynes and colleagues of the 1990's, as well as the mutiscale coarse-grained force matching method developed by the Voth group.

We thank the reviewer for pointing us to these papers. We have added references to Crippen and Snow 1990, Fujitsuka et al. 2004 from the Wolynes group, and Izvekov and Voth 2005. We have also added other references to bring the total to 94.
