# Supplementary material for: Differentiable molecular simulation can learn all the parameters in a coarse-grained force field for proteins
Source: PLoS One. 2021 Sep 2;16(9):e0256990. doi: 10.1371/journal.pone.0256990 (PMC8412298; doi:10.1371/journal.pone.0256990)
Supplement: Attachment — Submitted filename: response_to_reviewers_2.docx [file pone.0256990.s003.docx]

We have made the minor change as requested by reviewer 2 by removing the mention of the unused Langevin scaling factor. We believe the manuscript is now ready for publication.
